# *REEP4* variant analysis in blepharospasm and other neurological disorders

**DOI:** 10.3389/dyst.2024.12016

**Published:** 2024-02-07

**Authors:** Samira Saeirad, Mark S. LeDoux

**Affiliations:** 1 Department of Psychology, University of Memphis, Memphis, TN, United States; 2 Veracity Neuroscience LLC, Memphis, TN, United States

**Keywords:** blepharospasm, *REEP4*, Sanger sequencing, genetic variant, dystonia

## Abstract

**Introduction:**

In preceding work, a deleterious *REEP4* variant [GRCh38/hg38, NC_000008.11:g.22140245G>A, NM_025232.4:c.109C>T, p.Arg37Trp] was found to co-segregate with blepharospasm (BSP) in a large African-American pedigree. Other *REEP4* variants have been reported in genetic screening studies of dystonia. The *REEP4* paralogs, *REEP1* and *REEP2,* are associated with spastic paraplegia. The causal contributions of *REEP4* variants to dystonia and other neurological disorders remains indecisive.

**Methods:**

Sanger sequencing was used to screen subjects (*N* = 307) with BSP and BSP-plus dystonia affecting additional anatomical segments (BSP+) phenotypes for variants in *REEP4. In silico* tools were used to examine the deleteriousness of reported (ClinVar) and previously published *REEP4* variants.

**Results:**

No highly deleterious variant was identified in coding or contiguous splice site regions of *REEP4* in our cohort of 307 subjects. *In silico* analysis identified numerous deleterious *REEP4* variants in published screening studies of dystonia and several highly deleterious single nucleotide *REEP4* variants in ClinVar.

**Conclusion:**

Highly deleterious *REEP4* variants are rare in BSP and BSP+ phenotypes.

## Introduction

Blepharospasm (BSP) is a focal dystonia characterized by involuntary orbicularis oculi spasms that are usually bilateral, synchronous, and symmetrical [1]. BSP typically spreads to nearby craniocervical segments, including the lower face, masticatory muscles, and neck, resulting in segmental craniocervical dystonia [2, 3]. The term BSP-plus (BSP+) is used to describe individuals with BSP who experience further spread to additional anatomical segments [2–4].

A notable percentage of BSP probands have at least one 1st-degree relative with dystonia [5–8]. Defazio and co-workers [8] examined 233 relatives of 56 probands with isolated BSP and found a 1st-degree relative affected by BSP or other anatomical distribution of dystonia in 27%. Penetrance is approximately 20% in pedigrees with BSP [8, 9]. For comparison, penetrance of the classic ΔGAG mutation in *TOR1A* (DYT1) is 30%–40% [10]. Approximately 10% of subjects in published biorepositories of isolated dystonia report a relative with dystonia [11–15].

Even though late-onset isolated dystonias including BSP and BSP+ have a notable heritable component, large pedigrees adequately powered for linkage analysis are rare. Although rare cases of isolated BSP have been linked to *THAP1* mutations [16], the genetic underpinnings of this important focal dystonia remained largely unknown until identification of a deleterious co-segregating *REEP4* variant [GRCh38/hg38, NC_000008.11:g.22140245G> A, NM_025232.4:c.109C>T, p.Arg37Trp] in seven subjects with BSP or BSP+ from a 3-generation African-American BSP/BSP+ pedigree [17]. Follow-up screenings of large cohorts of patients with BSP and other forms of dystonia for *REEP4* variants is a logical and necessary next step in the study of BSP [18,19]. Additional *REEP4* variants have been identified in single cases of BSP, but to our knowledge, no other highly deleterious and co-segregating variants have been reported in other multiplex pedigrees.

REEP4, a member of the receptor expression-enhancing protein (REEP) family, contributes to nuclear pore formation [20]. Other dystonia-associated genes *(TOR1A* and *NUP62)* play critical roles in nuclear pore functions including nucleocytoplasmic trafficking [21, 22]. Spastic paraplegia (SPG) has been linked to mutations in other family members (REEP1/SPG31 and REEP2/SPG72). Although not yet reported in SGP31 and SPG72, dystonia is a phenotypic feature of many SPGs [23]. In aggregate, existing genetic, cellular, and clinical data suggest that *REEP4* is a candidate gene for dystonia and other neurological disorders.

## Materials and methods

The DNA samples used in this study were collected by the Dystonia Coalition (DC) and acquired from the Coriell Institute for Medical Research (Camden, New Jersey, United State). The DC is part of the Rare Diseases Clinical Research Network, which is funded by the National Institutes of Health and led by the National Center for Advancing Translational Sciences (NCATS). The DC is funded under a grant (U54NS116025) as a collaboration between NCATS and the National Institute of Neurological Disorders and Stroke. DNA analyses were approved by the University of Memphis Institutional Review Board. The cohort reported herein consisted of 307 subjects with BSP (*N* = 200) or BSP+ (*N* = 107) phenotypes. BSP+ phenotypes included subjects with BSP along with various combinations of lower facial, oromandibular and cervical dystonia. There were 224 females and 83 males with ages of acquisition ranging from 19 to 87 years. The median age at acquisition was 63 years with a mean ± standard deviation of 63.1 ± 11.0 years. Self-declared races included 259 whites, 1 Native American, 1 Pacific Islander, 11 Asians, 20 African Americans, 5 multi-racial, and 10 of other, not reported, or unknown race.

Genome assembly GRCh38.p14 served as the reference for primer design and variant annotation. Primers were designed to cover the coding regions of *REEP4* along with exon-intron boundaries (Supplementary Table S1). Sequencing also extended into proximal intergenic regions 5′ and 3′ to *REEP4.* Unidirectional Sanger sequencing was completed in the entire cohort of 307 subjects with BSP and BSP+ phenotypes. Bidirectional Sanger sequencing was used to confirm all identified variants.

ClinVar [24] and PubMed were analyzed for reported and published *REEP4* variants up to 15 July 2023. PubMed was interrogated with the search terms dystonia, blepharospasm, gene, genetics, mutation, genetic variant, Meige, and *REEP4*. The gnomAD V3.1.2 database was used to assess the frequency of these variants in a larger population [25, 26]. The v3.1.2 data set includes 76,156 whole genomes mapped to GRCh38/hg38k^1^.

CADD-Phred-scores [27, 28], MetaLR [29], and REVEL [30] were used to access variant deleteriousness. A CADD-Phred score of 10 indicates that the variant is among the 10% most deleterious in the genome, a score of 20 indicates that the variant is among the 1% most deleterious variant in the genome, and so on. Pathogenicity classification followed the standards set by the American College of Medical Genetics and Genomics [31], considering factors such as population data, variant databases, co-segregation, disease databases, and location of the variant within established functional domains of the encoded protein. Variants were classified by suggested terminology: “pathogenic,” “likely pathogenic,” “uncertain significance,” “likely benign,” and “benign.” The gnomAD v3.1.2 data set was examined for putative loss-of-function (pLoF) variants.

## Results

No highly deleterious variants were identified in our cohort of 307 subjects (Table 1). One variant (NM_025232.4: c.129T>A, NP_079508.2:p.Ile43Ile) resulted in an Exon 3 synonymous change from Isoleucine (Ile) to Isoleucine (Ile). Although mildly deleterious (CADD = 9.78), the c.129T>A variant was present in a higher percentage (7.7%) of gnomAD v3.1.2 alleles (z = −4.89, *p <* 0.0001). This variant is located within a ZNF263 binding site [32] (OREG1946722) and could play a role in gene expression. Five of the identified variants were non-coding. In comparison to our BSP/BSP+ cohort, all these variants were present in a higher percentage of gnomAD v3.1.2 alleles.

On 28 July 2023, ClinVar reported clinical significance for 93 variants (1 likely benign, 22 of uncertain significance, 4 likely pathogenic, and 66 pathogenic). Of the 66 pathogenic variants, 57 were duplications and 7 were deletions, all large structural variants affecting more than one gene. Of the 16 single nucleotide variants (SNVs), 1 was likely benign and 15 were of uncertain significance (Table 2). All SNVs were associated with an “inborn genetic disease” and deposited by a single submitter. Thirteen of these SNVs are highly deleterious to protein function with CADD scores >20 (Table 2). The variants are distributed across the encoded REEP4 from amino acid (aa) residues 16 to 226 (REEP4 Isoform 1 = 257 aa).

Three *REEP4* screening studies have been published to date. Hammer and colleagues [18] examined 132 patients (116 white) diagnosed with BSP or BSP+ phenotypes. A second study included 78 Han Chinese [19] and a third study from Hungary screened 47 nonrelated patients with BSP and 74 patients with cervical dystonia [32]. A total of 70 patients harbored a *REEP4* variant. A total of 19 variants were reported in the literature. The most common (*N* =27) was the synonymous variant (p.Ile43Ile) in Exon 3. Seven published variants included in Table 2 have CADD-Phred scores >20.0 but 3/7 have population prevalence rates above 0.01% (>1/10,000). We classified 10 of the 19 published variants as benign due to CADD-Phred scores <10 and high population prevalence rates. The p.Arg37Trp variant [17] was classified as likely pathogenic due to positive co-segregation in a large pedigree, CADD-Phred score >30, high REVEL and MetaLR scores, and low population prevalence. The other 8 variants were classified as “uncertain significance.”

A total of 11 unique unflagged pLoF *REEP4* variants is reported in 12 individuals within the gnomAD v3.1.2 database. Nine of these variants are in coding regions of *REEP4* (7 frameshift, 2 stop gained), and two are splice acceptors. CADD-Phred scores for these pLoF variants range from 25.7 to 45.0 with a mean of 31.9. The gnomAD v3.1.2 database does not provide Loss Intolerance probability (pLI). For the gnomAD v2.1.1 database, the pLI score for *REEP4* is 0.03.

## Discussion

Without comprehensive co-segregation analyses in pedigrees with dystonia and trio analyses in early-onset neurological disorders, it is not possible to convincingly ascribe pathogenicity to published and reported *REEP4* variants. Moreover, the study of dystonia genetics is compromised by subtle phenotypes, incomplete penetrance, possible pleiotropy, and the largely unexplored possibilities of recessive and oligogenic inheritance patterns. Assuming penetrance of 20% for a BSP-associated gene and BSP population prevalence of 50–100 cases per million [34], a possibly pathogenic variant should be seen in no more than 20/100,000 alleles. The variants tabulated herein that exceeded that threshold are unlikely to be causal in monogenic fashion.

In addition to lack of co-segregation analyses (e.g., phenotyping and genotyping all available family members) there are other obvious limitations to our screening study. First, Sanger sequencing will often miss exonic deletions and large structural variants. Second, our study was limited to BSP as driven by our previous identification of REEP4 p.Arg37Trp in an African-American family. It is possible that REEP4 plays a more important role in other forms of focal dystonia or spastic paraplegia. In this regard, a novel variant in *ATP5MC3* co-segregated with both dystonia and spastic paraplegia in a large multiplex pedigree [35]. Third, we have not characterized the biological effects of individual variants using cellular, invertebrate, or vertebrate model systems. *In silico* assessments of deleteriousness are informative but, in isolation, cannot establish causality.

The REEP family of REEP1–6 can be divided into subfamilies REEP1–4 and REEP5–6. The REEP1–4 subfamily can be separated into REEP1–2 and REEP3–4 groups based on structural/functional similarities [36]. While REEP1 and REEP2 are linked to HSP, REEP6 is associated with retinitis pigmentosa 77 [37]. REEP3 and REEP5 have not yet been associated with specific neuro-ophthalmological or general medical disorders. REEP4 protein is expressed in regions of the brain (basal ganglia and cerebellum) that play a role in the pathophysiology of dystonia^2^, and very few pLoF variants are included in the gnomAD v3.1.2 database. This information suggests a possible role for REEP4 in neuro-ophthalmological disorders. In this regard, the gnomAD v2.1.1 pLI score for single nucleotide variants must be recognized but interpreted with caution [38].

Several non-coding intronic and nearby intergenic *REEP4* variants were identified in our screening study but all of these were present at higher frequency in the gnomAD v.3.1.2 database. These differences in allele frequency are likely due to population stratification since the majority of Coriell DC samples were likely collected from only a few high enrolling sites whereas the gnomAD v.3.1.2 data was derived from more geographically diverse populations. One or more of these variants in isolation or a combination of variants (haplotype) could, in theory, play a role in *REEP4* expression. For instance, the intergenic variant NC_000008.11:g.22137633C>G is located within a regulatory region (OREG0018606) [39], binding site for transcription factors TFAP2C (OREG1194750), FOXP1 (OREG1608060), and CTCF (OREG1385204), and distal enhancer-like signature (dELS, EH38E2616119) [40].

In conclusion, more work is required to establish a convincing role for *REEP4* in dystonia and other neurological disorders. Of note, *REEP4* may make a more significant contribution to African American dystonia, whereas our work herein and other published screening studies of *REEP4* were focused on white Americans, Chinese, and Hungarians. Future studies should include patients with other forms of dystonia, more diverse racial and ethnic populations, and include phenotypic and genotypic assessments of other family members. Family studies with trio analyses are essential for understanding early-onset neurological disorders. Finally, inclusion of *REEP4* in commercial dystonia DNA panels would be of clear value to the scientific community.

## Supplementary Material

Supplementary Material

## Figures and Tables

**TABLE 1:** *REEP4* (GRCh38/hg38, NC_000008.11, NM_025232.4) variants identified with Sanger sequencing.

Variant	Number of subjects	Allele count	Homozygotes	Protein	gnomAD v3.1.2 allele frequency	CADD Phred-scaled	Clinical significance
NM_025232.4:c.418-43A>T (rs56401147)	73/307 (24%)	98/614 (16.0%)	15	NA (intronic)	34,782/152,086 (22.87%)	0.127	Benign
NC_000008.11:g.22137633C>G (rs79472476)	7/307 (1.63%)	7/614 (1.14%)	0	NA (downstream)	2,421/152,174 (1.59%)	2.76	Benign
NC_000008.11:g.22137584G>T (rs12676497)	57/307 (11.07%)	62/614 (10.1%)	5	NA (downstream)	29,058/151,988 (19.12%)	0.091	Benign
NM_025232.4:c.129T>A (rs35574275)	15/307 (4.89%)	15/614 (2.44%)	0	NP_079508.2:p.Ile43Ile	11,722/152,116 (7.71%)	9.78	Benign
NC_000008.11:g.22142150A>C (rs76026658)	9/307 (2.93%)	9/614 (1.47%)	0	NA (upstream)	5,989/152,200 (3.94%)	1.66	Benign
NM_025232.4:c.418– 25C>T (rs73549542)	7/307 (1.95%)	7/614 (1.14%)	0	NA (intronic)	4,029/152,238 (2.65%)	0.036	Benign

NA, not applicable

**TABLE 2:** REEP4 variants reported by NCBI’s ClinVar and PubMed.

Variant (accession)	Protein change	Condition (number of probands)	Clinical significance	gnomAD v3.1.2 (allele frequency)	CADD-Phred	MetaLR	REVEL	Reference
c.48G>C (SCV003984452.1)	p.Met16Ile	Inborn genetic disease (*N* =1)	Uncertain significance	2/152,270	23.1	0.55	0.24	[33]
c.101A>T (SCV003692342.1)	p. Glu34Val	Inborn genetic disease (*N* =1)	Uncertain significance	6/152,114	33.0	0.93	0.94	
c.116T>C (SCV003757666.1)	p. Met39Thr	Inborn genetic disease (*N* =1)	Uncertain significance	0	26.6	0.87	0.91	
c.118A>T (SCV003678640.1)	p. Met40Leu	Inborn genetic disease (*N* =1)	Uncertain significance	5/152,096	29.0	0.84	0.82	
c.160G>A (SCV003750691.1)	p.Val54Ile	Inborn genetic disease (*N* =1)	Likely benign	0	0.04	0.35	0.19	
c.250G>A (SCV003734663.1)	p. Ala84Thr	Inborn genetic disease (*N* =1)	Uncertain significance	12/152,152	24.1	0.85	0.69	
c.371G>A (SCV003558622.1)	p. Arg124Gln	Inborn genetic disease (*N* =1)	Uncertain significance	6/152,226	23.6	0.42	0.41	
c.382A>G (SCV003620006.1)	p. Ile128Val	Inborn genetic disease (*N* =1)	Uncertain significance	0	17.9	0.45	0.19	
c.436G>A (SCV003759714.1)	p. Gly146Ser	Inborn genetic disease (*N* =1)	Uncertain significance	29/152,214	23.6	0.54	0.30	
c.436G>T (SCV003974326.1)	p.Gly146Cys	Inborn genetic disease (*N* =1)	Uncertain significance	0	26.4	0.75	0.53	
c.473C>T (SCV003900529.1)	p. Ser158Phe	Inborn genetic disease (*N* =1)	Uncertain significance	1/152,242	22.5	0.60	0.25	
c.475A>T (SCV003730928.1)	p. Ile159Phe	Inborn genetic disease (*N* =1)	Uncertain significance	27/152,196	22.6	0.56	0.32	
c.583G>A (SCV003888930.1)	p. Asp195Asn	Inborn genetic disease (*N* =1)	Uncertain significance	22/152,192	21.7	0.33	0.19	
c.634G>C (SCV003759638.1)	p. Ala212Pro	Inborn genetic disease (*N* =1)	Uncertain significance	0	5.3	0.33	0.11	
c.662G>A (SCV004002432.1)	p.Arg221His	Inborn genetic disease (*N* =1)	Uncertain significance	14/152,208	23.4	0.59	0.49	
c.676C>T (SCV003893236.1)	p. Arg226Cys	Inborn genetic disease (*N* =1)	Uncertain significance	1/152,226	27.0	0.61	0.59	
c.109C>T (NM_025232.4)	p. Arg37Trp	BSP/BSP+ (*N* =1)	Likely pathogenic	3/152,168	31.0	0.960	0.77	[17]
c.-404_-401delAAGT (NM_025232.4)	NA	BSP (*N* = 3)	Uncertain significance	96/152,274	21.1	NA	NA	[19]
c.129T>A (NM_025232.4)	p.Ile43Ile	BSP (*N* = 4 + 23 = 27)	Benign	11,722/152,116	9.78	NA	NA	[18, 19]
c.303 + 137G>C (NM_025232.4)	NA	BSP (*N* = 5)	Benign	54/152,204	3.15	NA	NA	[19]
c.304-8G>T (NM_025232.4)	NA	BSP (*N* = 2)	Benign	124/152,174	0.025	NA	NA	[19]
c.446G>A (NM_025232.4)	p. Arg149Gln	BSP (*N* = 3)	Uncertain significance	501/152,226	29.6	0.4856	0.73	[19]
c.418-43A>T (NM_025232.4)	NA	BSP (*N* = 6)	Benign	34,782/152,086	0.13	NA	NA	[19]
c.*170_*188del (NM_025232.4)	NA	BSP (*N* = 8)	Benign	18,507/152,046	8.51	NA	NA	[19]
c.649C>T (NM_001316964.2)	p.Arg217Cys	BSP (*N* = 3)	Uncertain significance	1/152,226	9.34	0.66	0.20	[19]
c.*39C>T (NM_025232.4)	NA							
c.182 + 17C>T (NM_025232.4)	NA	BSP (*N* = 2)	Benign	174/152,206	0.22	NA	NA	[18]
c.312C>T (NM_025232.4)	p.Asp104Asp	BSP (*N* = 1)	Benign	738/152,206	1.18	NA	NA	[18]
c.418-12T>C (NM_025232.4)	NA	BSP (*N* = 1)	Benign	0	2.00	NA	NA	[18]
c.538C>T (NM_025232.4)	p. Arg180Trp	BSP (*N* = 1)	Uncertain significance	8/152,232	20.2	0.3472	0.34	[18]
c.539G>A (NM_025232.4)	p. Arg180Gln	BSP (*N* = 1)	Benign	2,415/152,220	4.28	0.2425	0.16	[18]
c.553 + 28T>C (NM_025232.4)	NA	BSP (*N* = 1)	Benign	2,427/152,206	3.95	NA	NA	[18]
c.661C>T (NM_025232.4)	p. Arg221Cys	BSP (3 subjects) (*N* =3)	Uncertain significance	703/152,212	24.0	0.4778	0.33	[18]
c.*95_*96insTCCACGTCTGTG (NM_025232.4)	p.Val251_Pro252insSerThrSerVal	BSP (*N* = 1)	Uncertain significance	195/152,226	17.0	NA	NA	[18]
c.538C>T (NM_025232.4)	p.Arg180Trp	Cervical dystonia (*N* =1)	Uncertain significance	8/152,232	20.2	0.44	0.34	[32]
c.734G>A (NM_025232.4)	p.Arg245Gln	BSP (*N* = 1)	Uncertain significance	30/152,110	21.2	0.38	0.12	[32]

NA, not applicable.

## Data Availability

The original contributions presented in the study are included in the article/Supplementary Material, further inquiries can be directed to the corresponding author.

## Footnotes

1
https://gnomad.broadinstitute.org/stats

2
proteinatlas.org
