# Supplementary material for: REEP4 variant analysis in blepharospasm and other neurological disorders
Source: Dystonia. Author manuscript; Available in PMC 2024 Sep 11. (PMC11390104; doi:10.3389/dyst.2024.12016)
Supplement: Supplementary Material [file NIHMS1982898-supplement-Supplementary_Material.pdf]

SUPPLEMENTARY MATERIAL

Table S1

REEP4 (GRCh38/hg38, NC_000008.11) primers for Sanger sequencing.

**Primer name**	**Sequence (5' → 3')**	**Locus**	**Product size (bp)**
*REEP4*_5'UTR-F	AATGCCTCAAATCATCGCGG	NC_000008.11: 22142479-459	596
REEP4-5'UTR-R	CTTGAAAGTTGCACGGAACC	NC_000008.11: 22141903-883	
REEP4-E1F	CCCACACGCTAATCTTTCCG	NC_000008.11:22142081-062	546
REEP4-E1R	TTCACTCAAGGGCTGGGAC	NC_000008.11:22141554-536	
REEP4-E1SF	CGGGTGGGAGCTGTAACTG	NC_000008.11:22141760-742	400
REEP4-E1SR	GTTCCTCCTCCCACTAGACG	NC_000008.11: 22141380- 361	
REEP4-E2F	CTTGGCAAGGTGGGAGTG	NC_000008.11: 22140756-739	250
REEP4-E2R	CCTCCCCACTCCACTCAG	NC_000008.11: 22140524-507	
REEP4-E3F	GGCTCCCTGGAATCTCTGG	NC_000008.11: 22140336-318	257
REEP4-E3R	GAACCTGTCACAGCACAAGG	NC_000008.11: 22140099-080	
REEP4-E4F	TGGCAGCAGAGATCGTTACA	NC_000008.11: 22140208-189	497
REEP4-E4R	ATGAGTGGCCATGCTCCA	NC_000008.11: 22139729-712	
REEP4-E5F	ACAACCAGTGGCTTCTCAGA	NC_000008.11: 22139611-592	434
REEP4-E5R	CAATGTGGCACCAACAGGAA	NC_000008.11: 22139197-178	
REEP4-E5SF	GAGCAGGGGTGAGAAGGTG	NC_000008.11: 22139314-296	474
REEP4-E5SR	CTTCCTCGAGCCTTGGGG	NC_000008.11: 22138858-841	
REEP4-E6F	TCACACCATGGCTTCCCC	NC_000008.11: 22138872-755	300
REEP4-E6R	CATGAGGGTGTGGGGAGC	NC_000008.11: 22138591-574	
REEP4-E7F	GAGGTCAGTGTGGGAGGAC	NC_000008.11: 22138641-623	392
REEP4-E7R	GGGCAGAGCAAGGCAATAAA	NC_000008.11: 22138269-249	
REEP4-E7SF	TCCTGCTGATGGTTAAGGGC	NC_000008.11: 22138365-346	398
REEP4-E7SR	CCATTTCAAGGGTGTGAGGC	NC_000008.11: 22137987-967	
REEP4-3'UTR-F	GGTCAGGTTTGCGAGTGTTT	NC_000008.11: 22138063-044	544
REEP4-3'UTR-R	CCAGATCCCAGACACTGCT	NC_000008.11: 22137538-521	
